# NLRP3 Inflammasome-Mediated Inflammation in Acute Pancreatitis

**DOI:** 10.3390/ijms21155386

**Published:** 2020-07-29

**Authors:** Ana Ferrero-Andrés, Arnau Panisello-Roselló, Joan Roselló-Catafau, Emma Folch-Puy

**Affiliations:** 1 Experimental Pathology Department, Institut d’Investigacions Biomèdiques de Barcelona-Consejo Superior de Investigaciones científicas (IIBB-CSIC), Barcelona, 08036 Catalonia, Spain; ana.ferrero@iibb.csic.es (A.F.-A.); arnau.panisello@iibb.csic.es (A.P.-R.); 2 Experimental Pathology Department, Institut d’Investigacions Biomèdiques de Barcelona-Consejo Superior de Investigaciones científicas (IIBB-CSIC), Centro de Investigación Biomédica en Red de Enfermedades Hepáticas y Digestivas (CIBEREHD), Institut d’Investigacions Biomèdiques August Pi i Sunyer (IDIBAPS), Barcelona, 08036 Catalonia, Spain; joan.rosello@iibb.csic.es

**Keywords:** inflammation, inflammasome, pancreatitis, immune system, interleukins, DAMPs, SIRS, NLRP3

## Abstract

The discovery of inflammasomes has enriched our knowledge in the pathogenesis of multiple inflammatory diseases. The NLR pyrin domain-containing protein 3 (NLRP3) has emerged as the most versatile and well-characterized inflammasome, consisting of an intracellular multi-protein complex that acts as a central driver of inflammation. Its activation depends on a tightly regulated two-step process, which includes a wide variety of unrelated stimuli. It is therefore not surprising that the specific regulatory mechanisms of NLRP3 inflammasome activation remain unclear. Inflammasome-mediated inflammation has become increasingly important in acute pancreatitis, an inflammatory disorder of the pancreas that is one of the fatal diseases of the gastrointestinal tract. This review presents an update on the progress of research into the contribution of the NLRP3 inflammasome to acute pancreatic injury, examining the mechanisms of NLRP3 activation by multiple signaling events, the downstream interleukin 1 family of cytokines involved and the current state of the literature on NLRP3 inflammasome-specific inhibitors.

## 1. Acute Pancreatitis: Initiating Events and Disease Progression

Acute pancreatitis (AP), a sudden inflammatory condition of the pancreas, is one of the leading causes of hospital admission for digestive diseases [1]. Nowadays, the most commonly used classification system for AP is the 2012 revision of the Atlanta classification, with definitions based on international consensus. This classification defines three degrees of severity: mild, moderately severe, and severe AP [2]. The mild form of pancreatitis is identified by the absence of organ failure. When organ failure is present within the first 24 h but resolves within 48 h the patient is classified as having moderately severe AP. If the patient develops persistent organ failure, they are classified as having severe AP. In such cases, acute lung injury is the most serious complication associated, since it accounts for the majority of deaths in untreated patients and in hospitalized patients who die within a week of the onset of AP [3]. Currently, apart from the supportive care, there is still no specific pharmacological therapy against this severe form of the disease. 

Although the etiology of AP is complex, almost all cases are due to a sterile factor that initiates pancreatic damage. The presence of gallstones obstructing the outflow of pancreatic fluid and excessive ethanol consumption causes AP in over 80% of cases regardless of the geographical distribution of the population [4]. Whatever the causative factor, in response to a predisposing insult, the pancreatic acinar cell undergoes a series of alterations that begin with the premature activation of pancreatic proteolytic enzymes, leading to cell damage and gland self-digestion. Injured acinar cells trigger an inflammatory process by releasing inflammatory cytokines and chemokines that mediate the recruitment and activation of circulating neutrophils and macrophages. The activated neutrophils release high concentrations of oxidants and cytotoxic agents, which further worsen the local pancreatic damage. As inflammation continues, neutrophils transmigrate across endothelial cells and cause the local inflammation to evolve into what is known as the systemic inflammatory response syndrome (SIRS), leading to a dysfunction of vital organs and, in some occasions, to organ failure and death [5]. 

These tissue-infiltrating neutrophils are the main producers of pro-inflammatory cytokines and chemokines, which are important mediators in the function of the innate immune system. As a major driver of the inflammatory response in AP, nuclear factor-kappa B (NF-κB) is a central transcription factor that regulates the expression of a large array of genes involved in inflammation. Among them, the cytokines interleukins (IL) 1β, 6, 8, 18 and tumor necrosis factor alpha (TNFα) or its soluble receptor, have been studied as markers of severity of AP [6]. Unlike other cytokines, cellular IL1β and IL18 are synthesized as precursor proteins and need to be cleaved to generate their biologically active forms. This process is dependent upon the assembly of a multi-protein signaling platform: the inflammasome.

### Pattern Recognition Receptors Contributing to Inflammation in Acute Pancreatitis

The innate immune and inflammatory cells express specialized receptors known as Pattern Recognition Receptors (PRRs), capable of recognizing molecules frequently found in pathogens (known as Pathogen-Associated Molecular Patterns—PAMPs), and endogenous molecules released from damaged cells, named Damage-Associated Molecular Patterns (DAMPs) [7]. Five different types of PRR have been identified to date expressed on the cell surface or in intracellular compartments, but they can be secreted into the blood stream and tissue fluids as well [8]. Transmembrane proteins toll-like receptors (TLRs) and C-type lectin receptors (CLRs) induce inflammatory responses through the recognition of their extracellular ligands. By contrast, cytoplasmic proteins including retinoic acid-inducible gene (RIG)-I-like receptors (RLRs), absence in melanoma 2 (AIM2)-like receptors (ALRs), and nucleotide-binding oligomerization domain (NOD)-like receptors (NLRs) recognize intracellular ligands. Following ligand recognition or cellular disruption, these receptors activate downstream signaling pathways resulting in the upregulation of pro-inflammatory cytokines and chemokines which are important in inflammatory and antimicrobial responses. Both ALRs and NLRs induce an inflammatory response starting with the formation of the inflammasome complex. 

TLRs and NLRs are major contributors to inflammation in AP. The deficiency of toll-like receptor 4 (TLR4) in mice has demonstrated the key role of this receptor for full tissue injury in AP, while its involvement in AP-associated acute lung injury appears to be important only when the disease is worsened by sepsis [9,10,11]. Toll-like receptor 9 (TLR9) also promotes the development of pancreatic injury, since TLR9-deficient mice had lower rates of edema, leukocyte infiltration and IL1β gene expression in the pancreas after cerulein administration. In the same study, mice treated with an antagonist of TLR9 after AP induction through taurolithocholic acid 3-sulphate (a more severe model of AP) reduced serum amylase, pancreatic necrosis and inflammatory cell infiltration in the systemic lungs [12]. Evaluating the role of the NLR nucleotide-binding oligomerization domain-containing protein 1 (NOD1) in pancreatitis pathogenesis, Tsuji and colleagues found that this signaling is essential to the development of pancreatitis; more interestingly, they showed that the activation of NOD1 stimulated by translocated commensal organisms is an indispensable element in sustaining and widening the inflammatory process in the pancreas [13]. More recently, the receptor for advanced glycation end-products (RAGE) has been recognized. RAGE was initially characterized and named for its ability to bind to advanced glycation end-products whose concentrations are known to increase in conditions such as diabetes, as well as during ageing. However, it is now widely accepted that RAGE functions as a PRR, since it binds with numerous PAMPS and DAMPS ligands. The interaction between RAGE and its ligands mainly results in a pro-inflammatory response, and can lead to stress events often favoring mitochondrial dysfunction or cellular senescence. In this regard, Kang et al. has provided evidence that RAGE mediates the nucleosome-induced AIM2 inflammasome activation in macrophages [14]. In addition, genetic deletion of RAGE protects against local and systemic lung injury in L-arginine-induced AP in mice.

Hence, pancreatic inflammation can activate these sensing components, TLR4, TLR9, RAGE and NOD1 expressed in pancreatic acinar, ductal and/or immune cells [10,11]. These components sense DAMPs and PAMPs as the first signals in the cells of the affected pancreas, inducing the formation of the NF-κB complex and its activation, which stimulates the expression of pro-IL1β and pro-IL18 [15]. The second signal that is activated by sensing components causes the assembly of inflammasome complex and effectors. Taken together, there is no doubt that while pancreatic inflammation may be initially triggered by intra-acinar events such as trypsinogen activation, it ultimately depends on the subsequent immune responses induced by the activation of components of the innate immune system.

## 2. Inflammasomes

In the early 2000s, Tschopp and colleagues introduced the concept of the inflammasome, a group of cytosolic multi-protein complexes expressed in myeloid cells responsible for activation of inflammatory processes as part of the innate immune system [15]. Inflammasomes consist of an upstream sensor protein (belonging to the NLR or the ALR family), and an adaptor protein—the apoptosis-associated speck-like protein containing a caspase recruitment domain (ASC). ASC serves as a bridge, connecting the sensor to the downstream effector, the cysteine protease caspase-1 (formerly known as IL1β converting enzyme, ICE) [12]. Active caspase-1 cleaves the precursor cytokines pro-IL1β and pro-IL18, generating their biologically active forms (IL1β and IL18 respectively). Active caspase-1 is also able to induce an inflammatory form of cell death known as pyroptosis, which involves cell swelling, membrane rupture, and release of the cytoplasmic content into the extracellular space [16]. This pathway is known today as the canonical inflammasome. By contrast, more recently, Kayagaki et al. described a new non-canonical inflammasome pathway that is independent of caspase-1 [17]. 

### 2.1. The NLRP3 Inflammasome 

Since the discovery of the founding member of the NLR family, NOD1, 22 distinct NLR proteins have been identified in humans and 34 in mice [18]. To date, the best characterized NLR inflammasome is the NLR pyrin domain-containing protein 3 (NLRP3), also known as cryopyrin or NALP3. NLRP3 is a cytosolic receptor expressed in monocytes, neutrophils, dendritic cells, lymphocytes, osteoblasts and epithelial cells [19]. It is composed by three domains: a carboxy-terminal leucine-rich-repeat (LRR), a central nucleotide-binding and oligomerization (NACHT) domain, and an amino terminal pyrin domain (PYD). This last domain allows the joining to the pyrin domain of ASC through PYD–PYD interactions, and then the assembly with pro-caspase-1 by the caspase activation and recruitment domain (CARD)–CARD interactions, whose active form caspase-1 initiates the activation of proinflammatory cytokines IL1β and IL18 [16]. The ASC domain acts as a cell-to-cell communication signal and so can intensify the inflammasome response, processing IL1β in the extracellular space and sending a danger signal. Every domain has a function inside the inflammasome assembly: LRR triggers the danger signal response and the autoregulation of the inflammasome, the PYD domain recruits the adaptor molecule ASC, and NACHT carries out oligomerization and hydrolyses the ATP binding to it. This domain has ATPase activity, which is important for regulating the activation of inflammasome [20]. 

The NLRP3 inflammasome is considered a general sensor of cellular damage that responds to both PAMPS and DAMPs. However, it is the recognition of DAMPS that confers particular importance on NLRP3 in the context of the sterile inflammatory responses observed in many human diseases such as AP. Here, we present a comprehensive review of current developments in the study of the mechanism of action of the NLRP3 inflammasome in acute inflammation of the pancreas.

### 2.2. Signals of Action of the NLRP3 Inflammasome in the Immune Response

Canonical NLRP3 inflammasome is activated in two parallel and independent steps: priming, and activation. The first step is regulated by innate immune signaling in which a stimulus joins to TLR, NLR or cytokine receptors stimulating NF-κB activation, and this produces the upregulation of the mRNA and protein expression of NLRP3 and pro-IL1β [21]. Toll-like receptor (TLR)-adaptor molecules myeloid differentiation primary response 88 (MyD88) and toll/IL1 receptor homology-domain-containing adaptor-inducing interferon-β (TRIF) mediates this step in response to TLRs receptors. All TLRs except TLR3 can activate the MyD88-dependent pathway which results in the transcription of pro-inflammatory genes through the activation of NF-kB. Priming signal upregulates the expression of NLRP3, which is thought to exist at concentrations that are inadequate for initiating inflammasome activation under resting conditions, and pro-IL1β, which is not constitutively expressed. In contrast, priming signals do not appear to affect the expression levels of ASC, pro-caspase-1, or pro-IL18. 

Recently, several studies have provided strong evidence that the priming step is not limited to the transcriptional upregulation of NLRP3, since post-translational modifications (ubiquitination and phosphorylation, sumoylation and ribosylation) of NLRP3 protein also play significant roles in NLRP3 inflammasome regulation [22,23,24,25]. After priming, canonical NLRP3 inflammasome activation requires a second signal (activation signal) that results in NLRP3 inflammasome oligomerization, leading to caspase-1 activation and, in turn, IL1β and IL18 processing and release. This step depends on different stimuli: re-localization of NLRP3 to the mitochondria, releasing of mitochondrial (mt) reactive oxygen species (ROS) or DNA (mtDNA) or cardiolipin into the cytosol, increasing potassium efflux, the release of cathepsin from damaged lysosomal membranes, extracellular ATP, pore-forming particulate matter, pathogen-associated RNA and bacterial and fungal toxins and components [26]. Independently of IL1β maturation, caspase-1 activation also promotes pyroptosis through the cleavage of the pore-forming protein gasdermin-D (GSDM-D). Pyroptosis then promotes the release of additional cytosolic proteins, such as high mobility group box 1 (HMGB1), a pro-inflammatory mediator that plays a significant role in the pathogenesis of several inflammatory diseases such as AP [27]. 

Besides canonical NLRP3 inflammasome activation, a non-canonical caspase-11-dependent NLRP3 activation has been characterized, mainly in relation to Gram-negative bacteria [28]. In the first step, Gram-negative bacteria activate the TLR4–MyD88 and TRIF pathways, leading to nuclear translocation of NF-κB, which in turn promotes the transcription of IL1β, IL18, and NLRP3 as well as interferon regulatory factor (IRF)-3 and IRF7 genes. Subsequently, the IRF3–IRF7 complex leads to activation of the JAK/STAT pathway and the consequent transcription of caspase-11 gene in mice (or its human analogues caspase-4 and caspase-5) [29]. Conversely, when LPS is in abundance or has entered the cytosol through other processes, caspase-11 binds to this intracellular LPS independently of TLR4 (the well-known extracellular receptor for LPS). Direct interaction between the lipid A portion of LPS and the CARD domain of caspase-11 catalyzes the oligomerization of the caspase-11-LPS complex. This complex induces pyroptosis through the cleavage of GSDM-D and leads to a leakage of its N-terminal domain, forming pores in the plasma membrane through which IL18 and IL1β mature forms are released outside the cytosol. 

Both processes of canonical and non-canonical NLRP3 inflammasome activation occur independently. However, non-canonical caspase-11 enhances canonical caspase-1 processing and IL1β/IL18 production in the presence of specific stimuli (e.g., cholerae toxin or *E. coli*) [30]. Additionally, another protease required in transcriptional priming and activation of canonical and non-canonical NLRP3 inflammasome is caspase-8, best characterized as an initiator caspase involved in death receptor-mediated apoptosis in response to external stimuli [31]. 

## 3. The NLRP3 Inflammasome in the Pathogenesis of Acute Pancreatitis and Associated Lung Injury

Patients with AP exhibit elevated serum levels of pro-inflammatory cytokines such as IL1β, TNFα, IL6 and IL18 [32,33]. As we noted before, the precursor form of IL1β and IL18 cytokines is converted into an active form via the NLRP3 inflammasome, and so the NLRP3 inflammasome is likely to play an important role in AP. For that very reason, Hoque and Mehal elucidated the molecular mechanisms of NLRP3 inflammasome contributing to the initial inflammation in AP and progression [12]. They demonstrated that the NLRP3 inflammasome is notably activated during AP and that components of this inflammasome are required for full pancreatic injury. In an experimental model of cerulein-induced AP in mice, the absence of caspase-1, ASC or NLRP3 substantially reduced edema and inflammation. Furthermore, in a more severe model of AP in mice, TLR9 inhibition decreased both pancreatic IL1β expression and lung inflammation. Another study using NLRP3-deficient mice or the NLRP3 inhibitor INF-39 found suppression of the maturation and release of IL1β and further prevention of the inflammatory cascade in a cerulein plus LPS-induced AP model [34]. 

The involvement of TLR4 in the initiation of the disease was also demonstrated by Hoque using the metabolic intermediate lactate to block this receptor [35]. In a mouse model of cerulein plus LPS-induced AP, the administration of lactate reduced TLR4-mediated activation of NLRP3 inflammasome via Gi-protein-coupled receptor 81 signaling. This finding is concordant with clinical studies showing an anti-inflammatory effect of Ringer’s lactate solution for fluid resuscitation in patients with AP [36,37]. Other TLR4 modulators (e.g., a natural activator of cofactor NAD^+^ or carbon monoxide) have been used as well, demonstrating a clear role of the NLRP3 inflammasome in AP [38,39]. 

Further definitive support for the involvement of NLRP3 inflammasome in the development of lung injury secondary to pancreatitis comes from a recent study in exosome research. In that study, the plasma-derived exosomes triggered NLRP3 inflammasome activation and pyroptosis in alveolar macrophages, thus leading to pulmonary dysfunction in the progression of pancreatitis [40]. This study is the first to describe the process of pyroptosis as a driver of AP-associated lung injury. Notably, depletion of exosomes only partially abrogated the pyroptosis-inducing effect of AP-conditioned plasma indicating that, besides exosomes, other components are probably needed to promote alveolar macrophage pyroptosis in the progression of AP.

Until now, there has been little clinical information supporting the experimental results regarding inflammasome activation in AP. A study from Algaba-Chueca et al. identified increased levels of AIM2 and NLRP3 inflammasomes and derived IL1β and IL18 in the early course of AP [41]; furthermore, AIM2 expression was increased in patients who developed moderate or severe AP. In a very recent study, a rise in free ASC and IL18 was found in parallel with increased AP severity, suggesting a clear correlation between inflammasome activation and the progression of systemic complications in patients suffering from this disease [42]. In this elegant study, the authors propose a new model to understand the progression of inflammation associated with AP in which both SIRS and compensatory anti-inflammatory response syndrome (CARS) phases are initiated early during AP and progress in parallel. This proposal challenges the previously assumed sequential activation of SIRS and CARS. Interestingly, both pathways are regulated by the NLRP3 inflammasome-derived IL18. This cytokine plays a pivotal role by inducing a pro-inflammatory response of the innate immune system and acting as a Th2-cell mediator for the adaptive immune system. 

### 3.1. NLRP3 Inflammasome Activation in Acute Pancreatitis

#### 3.1.1. Extracellular DAMPs

We emphasize that, differently from other inflammasomes which only respond to few specific PAMPs, NLRP3 Inflammasome is activated by a wide variety of stimuli. Among them, DAMPs have attracted increased interest because of their impact in the pathogenesis of many human diseases. Apart from immune cells, several types of non-immune cells, such as epithelial cells, endothelial cells and fibroblasts, can be activated by DAMPs. 

HMGB1 is a nuclear molecule constitutively expressed in nearly all cells. Its presence seems indispensable for life, since HMGB1-deficient animals die shortly after birth [43]. As a nuclear protein, HMGB1 acts as a DNA chaperone and facilitates the binding of transcription factors to DNA. Under a variety of stressful situations, however, HMGB1 translocate to the cytosol where it sustains autophagy, and then it is released into the extracellular space. Outside the cell, HMGB1 may function as a DAMP with the ability to trigger inflammatory mediators [44].

**Figure 1:**
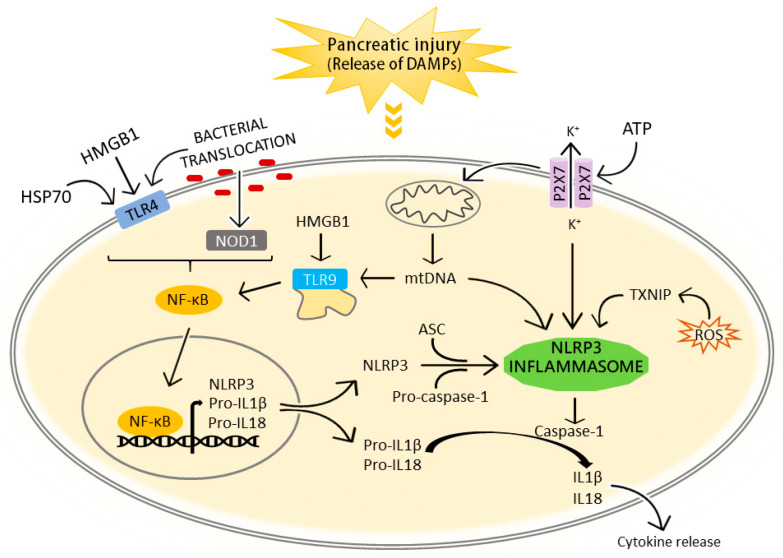
Schematic diagram illustrating the mechanisms of NLRP3 inflammasome activation during acute pancreatitis. DAMPs and gut bacteria have been recognized for their crucial role in the initial onset of pancreatic inflammation. Prototypical DAMPs derived from pancreatic injured cells include the HMGB1, HSP70 and purine metabolites, such as ATP. HMGB1, HSP70 and the translocation of intestinal bacteria can act through TLR4 in acute pancreatitis stimulating the NF-κB activation, and further upregulation of the mRNA and protein expression of NLRP3, pro-IL1β and pro-IL18. Moreover, the stimulation of intracellular NOD1 by translocated bacteria is an indispensable element to sustain the inflammatory process in the pancreas. Extracellular ATP, released by damaged cells, interacts with P2X7 inducing mitochondrial dysfunction and intracellular K^+^-depletion which results in NLRP3 assembly, caspase-1 activation, maturation of pro-IL1β and pro-IL18 and IL1β and IL18 secretion. Some intracellular damage-associated events have also been suggested to initiate NLRP3 inflammasome assembly in acute pancreatitis: mitochondrial DNA directly binds to NLRP3, and ROS production detaches TXNIP from thioredoxin and enables NLRP3 activation. Additionally, TLR9 senses intracellular HMGB1 and mtDNA with subsequent activation of NF-κB. LPS, lipopolysaccharide; HSP70, heat shock protein 70; HMGB1, high mobility group box 1; TLR4, toll like receptor 4; TLR9, toll like receptor 9; ROS, reactive oxygen species; ATP, adenosine triphosphate; NLRP3, NLR pyrin domain containing protein 3; ASC, caspase recruitment domain; NOD1, nucleotide-binding oligomerization domain 1; NF-kB, nuclear factor-kappa B; mtDNA, mitochondrial DNA.

Serum levels of HMGB1 are elevated in several inflammatory diseases, including sepsis, mechanical trauma, acute myocardial infarction, acute respiratory distress syndrome, hepatic injury, rheumatoid arthritis and stroke [45]. During AP, damaged pancreatic acinar cells release different intracellular contents, including DAMPs, which in turn promote NLRP3 inflammasome activation and trigger the inflammatory response (Figure 1). The circulating HMGB1 levels in AP are significantly increased and positively correlate with the severity of the disease both in humans and in experimental animal models [46,47,48,49]. Additionally, the use of antibodies against HMGB1, the pharmacological blockade or delayed therapeutic delivery confers protection against injury in experimental AP [50,51,52,53,54].

Extracellular HMGB1 signaling induces and enhances sterile inflammatory responses through TLR4 and TLR9 [55]. TLR4 is a well-known LPS-recognizing receptor, but responds to several DAMPs as well. In this sense, HMGB1 activates the TLR4-mediated NF-κB signaling pathway to induce pancreatic injury in AP while this pancreatic injury is significantly reduced in TLR4-deficient mice [56]. Extracellular HMBG1 it is also known to complex with nucleic acid DAMPs released from necrotic cells and promote TLR9 recognition [57]. A role for TLR9 as sensor of DAMPs in AP has been established by Hoque et al. since TLR9 inhibition could decrease both pancreatic IL1β expression and lung inflammation in experimental AP [12]. The potential mechanism responsible for these phenomena is that the increased release of mitochondrial DNA (mtDNA) and nuclear DNA (nDNA) during pancreatic injury activates TLR9 as well as NLRP3 inflammasome pathways. Conversely, a more recent study demonstrated that HMGB1 is involved in the activation of another inflammasome, the AIM2 inflammasome, mediated by the receptor RAGE [14]. Thus, mice lacking RAGE did not develop an inflammatory response after AP induced by L-arginine or cerulein. 

Heat shock proteins (HSP) are highly conserved proteins found in all prokaryotes and eukaryotes which intracellular levels markedly increase in front of a wide variety of stressful and biological alterations [58,59]. Heat shock protein 70 (HSP70) has been well characterized as an intracellular molecular chaperone although, in the recent years, there has been a rise in its role as a DAMP in the extracellular environment [60,61]. For instance, extracellular HSP70 (eHSP70) has been found to modulate the immune response via the MyD88/IRAK/NF-κB signal transduction pathway through TLRs and CD14 interaction [61,62]. 

The expression of HSP70 is upregulated in experimental AP and contributes in heat stress induced protection [63]. Further evidence for the functional significance of HSP70 in the modulation of AP damage came from a clinical study that revealed that the HSP70.2 gene polymorphism expression was linked to the severity of pancreatitis [64]. To date, a role for eHSP70 in AP has been reported in one study where the administration of recombinant HSP70 in mice aggravated cerulein-induced AP in a TLR4-dependent manner [65]. Additionally, a recent study from Somensi et al. suggested that eHSP70 is able to induce NF-κB gene activation through RAGE ligation and ERK signaling pathway in a lung carcinoma cell line [66]. In this context, it would be interesting to evaluate if eHSP70 could also modulate AP-associated immune response via RAGE interaction.

Although ATP is well recognized as a source of high energy phosphate bonds to support cellular metabolism, once is released from cells following cellular damage, it acts as a DAMP signal. Cellular necrosis and apoptosis trigger the release of ATP and other nucleotides into the extracellular space. These can prompt pro-inflammatory immune responses via cell-surface P2X7 purinergic receptor [67,68]. In a model of cerulein-induced AP in mice, extracellular ATP binds to P2X7, one of the most potent activators of the NLRP3 inflammasome, and results in NLRP3 assembly, caspase-1 activation and IL1β secretion [12]. In addition, the neutralization or blockage of P2X7 limited systemic injury but did not ameliorate the local pancreatic injury in different experimental models of AP of varying severity [69]. 

#### 3.1.2. Bacterial Translocation

Infection and bacterial colonization of the inflamed pancreas occurs in severe forms of AP being one of the most feared complications of the disease [70]. Bacterial translocation in AP is supported by studies showing that antibiotic treatment reduces or prevents pancreatic inflammation, infection, and mortality in various experimental pancreatitis models [13,71,72,73] and limiting Gram-negative colonization of the digestive tract significantly reduce mortality in humans with severe AP [74,75]. 

TLR4 activation is one of the mechanisms by which bacterial translocation may account for the development of severe experimental AP [9]. As in the case of TLR4, commensal organisms as well as pathogens can induce NOD1 signaling. In this sense, the simultaneous administration of low doses of cerulein with a NOD1 ligand (neither of which is able to induce pancreatitis on its own) in mice demonstrated that NOD1 signaling is essential to the development of pancreatitis [13]. Moreover, NOD1-deficient mice were almost completely resistant to cerulein-induced AP.

### 3.2. The IL1 Family of Cytokines as Effectors of the NLRP3 Inflammasome: Their Role in Acute Pancreatitis

The IL1 superfamily of cytokines are important regulators of innate and adaptive immunity, playing key roles in the host defense against infection, inflammation, injury, and stress. IL1α and IL1β were the founding members of this family of cytokines which comprise eleven members, the others being IL18, IL33, IL36α, β and γ, IL1 receptor (IL1R) agonist, IL36Ra, IL37, and IL38 [76]. This section highlights recent advances in the understanding of the molecular and cellular mechanisms of AP associated with NLRP3 inflammasome-regulated IL1 cytokines, focusing particularly on IL1, IL33, and IL18.

#### 3.2.1. IL1β

Among all the IL1 cytokines, IL1β is the one that is most active in mediating inflammation in processes of sterile necrosis, an important event in AP [77]. Indeed, the discovery of the inflammasomes was an essential step in improving our understanding of the molecular mechanisms of IL1β-mediated inflammation, since this multi-protein complex plays a critical role in the regulation of IL1β maturation. 

It is widely recognized that the development of AP depends on pro-inflammatory cytokine responses secreted by leukocytes accumulated in the inflamed pancreas [78]. TNFα and IL1β are considered primary cytokines mediating early-phase inflammation and propagation to extra pancreatic tissues during AP. As mentioned, there is significant evidence that inflammasomes and IL1β maturation are required for the induction of inflammation in AP [79]. IL1β is transcribed by monocytes, macrophages, and dendritic cells following TLR activation by PAMPs or by the cytokines TNFα, IL18, IL1α or IL1β itself. In fact, IL1β self-induction is a part of the mechanism of autoinflammation [80]. Thus, it is important to stress that activation of IL1β can also occur via alternative mechanisms independently of the inflammasomes. For instance, neutrophil-derived serine proteases and pathogen-released enzymes can also process and activate IL1β, and these processes have important effects during inflammation and infection [81]. 

#### 3.2.2. IL18

IL18 is best known for its capacity to induce Interferon γ (IFNγ). It is expressed by macrophages, epithelial cells and dendritic cells and is stored in the cytoplasm [82]. As occurs with IL1β, the NLRP3 inflammasome-caspase-1-dependent mechanism of IL18 maturation and release is the most common, although some caspase-1-independent mechanisms of cleavage have also been reported. IL18 functions by ligation of IL18 receptors (IL18R) α and β, and the complex recruits MyD88. Then, MyD88-induced events result in the activation of NF-κB and mitogen-activated protein kinase (MAPK) via association with the signal adaptors IL1R-associated kinase (IRAK) 1–4 and tumor necrosis factor receptor-activated factor 6 (TRAF6) respectively [83]. 

In humans, serum levels of IL18 have been widely associated with the pathogenesis of AP and are known to correlate with disease severity [6,84,85,86]. Additionally, induced IL18 levels have been reported in pancreas and lungs of rats with AP [87,88]. These findings contrast with those of study showing that genetic deficiency of IL18 in mice resulted in significantly exacerbated pancreatic injury in a model of cerulein-induced AP [89]. The controversy could be due to differences in the model and in the severity of the disease. Notably, IL18 plus IL12 administration is a well-characterized model of experimental pancreatitis in obese mice, emphasizing the importance of IL18 in the pathophysiology of the disease [90]. 

The involvement of inflammasome-derived IL18 in AP was first indicated in a 2007 study in which the therapeutic effects of caspase-1 inhibitors in acute lung injury associated with AP were related with the inhibition of IL1β and IL18 [88]. Sendler and co-workers have completed the picture in a very recent study which suggests a clear correlation of inflammasome activation and AP severity [42]. IL18 deficiency in mice resulted in a complete absence of Th2 response and a reduction of Th1 after the onset of AP. These findings underline the importance of IL18 for T-cell activation during AP and challenges the notion of the sequential activation of SIRS and CARS, proposing that both responses occur in parallel during severe AP. 

#### 3.2.3. IL33

IL33, the latest addition to the IL1 superfamily, was discovered over a decade ago and is now clearly defined as a key component of innate and adaptive immune responses. IL33 is constitutively expressed in epithelial and endothelial cells, and following translation is stored as a biologically active molecule in the nucleus where it binds to chromatin [91]. When cells undergo necrosis or are stressed, nuclear IL33 is immediately available to act as an early signal of damage. Unlike other members of the IL1 family, IL33 does not require processing through an inflammasome in order to achieve its biological activity and, in fact, it is inactivated by caspase cleavage [92]. However, cleavage by neutrophil elastase and cathepsin G proteases, which are found in the microenvironment during inflammation, can increase its potency [93]. Upon release of IL33 by DAMPs and PAMPs, IL33 specifically binds to the ST2 receptor in target cells and undergoes conformational changes resulting in the recruitment of IL1 receptor accessory protein (IL1RAcP) forming a ternary complex. In most cell types, and similar to IL18, IL33 signaling activates the MyD88/IRAK/TRAF6 axis resulting in the activation of downstream NF-κB and MAPK signaling pathways [94]. 

Although several lines of evidence demonstrate a role for IL33 in fibrogenesis during chronic pancreatitis [95], investigations into the potential function of IL33 in the pathogenesis of AP are limited. As in IL1β and IL18, serum levels of this cytokine are increased in patients at the early stage of AP and correlate with AP severity. The same findings have been reported using experimental animal models of AP [96,97,98]. Thus, in a bile duct ligation model of AP, exogenous IL33 administration exacerbated pancreatic inflammation and mast cell degranulation [98]. Similarly, in experimental sodium taurocholate-induced AP, IL33/ST2 signaling was found to be a major mediator of the disease through TNFα [96]. Conversely, data from other experimental studies in IL33 receptor ST2 deficiency suggested a protective role of IL33 in AP [99,100]. Although the effects of IL33 have been shown to be either pro- or anti-inflammatory depending on the disease and the model, many questions about its potential dual effect in AP remain to be resolved. Further additional studies are clearly needed on this issue and also on the role of inflammasome in IL33 processing and release during AP, which is not currently supported in the literature. 

## 4. Inhibitors of the NLRP3 Inflammasome

The association of the NLRP3 inflammasome with a myriad of human diseases has encouraged researchers to search for effective inhibitors of this multi-protein complex. To date, a wide variety of NLPR3 inflammasome inhibitors have been studied in experimental models of human diseases, but clinical application of this knowledge remains limited.

### 4.1. The Clinical Application of NLRP3 Inflammasome Inhibitors: the IL1 Antagonists

The currently available clinical treatment for NLRP3-related diseases involve the agents that target IL1β, including the recombinant IL1 receptor antagonist anakinra, the soluble decoy IL1β receptor rilonacept, and the neutralizing IL1β antibody canakinumab. The effectiveness of these inhibitors in AP has been demonstrated in the case of anakinra which significantly decreased cerulein-related pancreatic tissue injury and pancreatic apoptosis in rats [101]. 

Anakinra is a recombinant form of the IL1R antagonist approved for the treatment of rheumatoid arthritis in 2001. However, its clinical indications were extended, in 2003, to other conditions such as cryopyrin-associated periodic syndrome (CAPS), a group of rare inherited autoinflammatory diseases generally caused by autosomal-dominant mutations in the NLRP3 gene [102]. Because of the safety and rapid onset of action, IL1 inhibition with anakinra occupies an important position in IL1 therapeutics [103]. The development of Rilonacept was later introduced in 2008. Rilonacept is a fusion protein that incorporates the extracellular domains of the IL1R components required for IL1 signaling and the Fc portion of immunoglobulin G [104]. As anakinra, it was indicated for the treatment of CAPS caused by dysregulated IL1 production.

The third therapeutic option, canakinumab, is a humanized monoclonal antibody against IL1β which does not cross react with other members of the IL1 family [105]. Canakinumab was first authorized by the FDA in 2009 for the treatment of CAPS and active systemic juvenile idiopathic arthritis [106]. In 2016, it received approval as first-line treatment for TNF receptor associated periodic syndrome, familial Mediterranean fever and mevalonate kinase deficiency which are also linked to the activation of the pyrin inflammasome. 

All agents reduce or even resolve clinical symptoms, decrease the biochemical activity markers associated and improve quality of life in CAPS patients [107]. In addition, they are currently applied in other inflammatory disorders and as antitumor drugs—as one might expect, given that inflammation is a hallmark of early tumorigenic events.

### 4.2. NLRP3 Inflammasome Inhibitors in Acute Pancreatitis

The evidence of NLRP3 inhibition in AP comes from experimental data since no clinical studies have yet explored the benefits of the inhibition of IL1β or other inflammasome components. Here, we present an overview of the inhibitors of the inflammasome pathway in experimental AP reported to date (Table 1).

#### 4.2.1. Sulphonylureas Drugs

Glyburide, also known as glibenclamide, is an NLRP3 inhibitor of the class of sulphonylureas drugs widely used for the treatment of type 2 diabetes. Glyburide is not a direct inhibitor of the NLRP3 inflammasome since it interferes with signaling events upstream of the NLRP3 assembly. Specifically, it blocks ATP-sensitive potassium (K^+^) channels on the pancreatic β cell membrane preventing a cellular efflux of K^+^, a known danger signal for inflammasome activation [121]. The administration of glyburide in a mouse model of cerulein AP significantly reduced serum levels of IL6, lipase, and amylase and lowered the IL1β release in cultured peritoneal cells treated with LPS [108]. 

MCC950 is a diarylsulfonylurea-containing compound considered one of the most potent and selective inhibitors of the NLRP3 inflammasome [122]. MCC950 functions by blocking both canonical and non-canonical NLRP3 inflammasome activation by abrogating ASC oligomerization instead of K^+^ efflux, Ca^2+^ flux or NLRP3–ASC interactions. Notably, MCC950 had no inhibitory effect on the activation of other inflammasomes complexes such as AIM2, NLRC4, or NLRP1 [123]. MCC950 has previously been involved as a potential treatment in an increasing number of inflammatory diseases, including atherosclerosis, nonalcoholic fatty liver disease, Alzheimer’s disease and myocardial infarction [124]. The study by Sendler et al. is the only one to have assessed the use of MCC950 in experimental AP [42]. The therapeutic administration of this small molecule lowered IL18 levels and significantly reduced disease severity and systemic injury. 

#### 4.2.2. Natural Products from Plants and Fungi

A large number of compounds of plant origin, including polyphenols, terpenes, alkaloids, glycosides, quinones and flavonoids, have been explored for the treatment of a variety of pancreatic disorders including diabetes and pancreatitis. Here, we describe a large number of NLRP3-blocking compounds that may be able to deal with the inflammatory challenge of AP. 

Emodin is an anthraquinone derivative (1,3,8-trihydroxy-6-methyl-anthraquinone) that can be isolated from rhubarb, buckthorn, and Japanese knotweed and has proven antitumoral, anti-inflammatory and immunomodulatory effects [125]. Previous experimental studies have confirmed that emodin alleviates lung injury associated to AP [126,127], but the involvement of NLRP3 inflammasome on the protective effects of emodin have been recently investigated. Zhang et al. found that this natural compound delayed the progression of AP through P2X7/NLRP3 signaling pathway inhibition, thereby improving the associated systemic inflammation [109]. Moreover, in the same experimental model of severe AP, emodin exerted its protective effect by inhibiting NLRP3 inflammasome activation via Nuclear factor erythrocyte-2 associated factor 2 (Nrf2) pathway [110]. 

Danshensu is a water-soluble ingredient of danshen, an active constituent of Salvia miltiorrhiza generally known to exert cardioprotective function in myocardial ischemic injury [128,129]. Danshensu has been reported to attenuate LPS-induced inflammatory responses and exhibit anti-oxidative effects in cultured macrophages [130]. In a mouse model of cerulein-induced AP, Danshensu directly suppressed the pancreatic activation of the NLRP3 inflammasome and NF-kB and STAT3 signaling pathways [111].

Fraxinellone is a natural component isolated from the plant Dictamnus dasycarpus and has been reported to exert anti-inflammatory activity through the suppression of NF-κB in macrophages [131]. Its inhibitory effect on the activation of the NLRP3 inflammasome cascade has been observed in three different experimental models of AP [112]. The treatment of mice with fraxinellone significantly attenuated the severity of AP by inhibiting the pancreatic activation of multiple inflammasome molecules such as NLRP3, PY-CARD, caspase-1, IL18, and IL1β. Additionally, fraxinellone inhibited the infiltration of macrophages and neutrophils into the pancreas through the suppression of inflammasome signaling. 

Withaferin A is an alkaloid isolated from ginseng (Withania somnifera), with pharmacological properties ranging from antitumoral to anti-diabetic and anti-inflammatory effects [132,133,134]. Withaferin A effectively suppresses the sustained activation of endoplasmic reticulum stress and inhibits NLRP3 inflammasome activation during cerulein-induced AP in mice [113].

Rutin is a flavonoid found in certain vegetables and fruits. In an L-arginine-induced AP model in mice, rutin decreased the pancreatic injury but also furthered catalase and superoxide dismutase antioxidant activities [135]. Rutin treatment in rats with cerulein-induced AP and fed on ethanol reduced pancreatic inflammation and modulated the NLRP3 inflammasome by attenuating the expression of ASC, caspase-1 and IL1β [114].

Sulforaphane, a naturally occurring isothiocyanate, is mainly present in vegetables and plays an important role in the maintenance of cellular redox balance [136]. Sulforaphane is the most widely studied member of isothiocyanates, in both in vivo and in vitro models of different diseases, (mainly diabetes and cancer). In a mouse model of cerulein-induced AP, sulforaphane protected from pancreatic damage by exerting antioxidant and anti-inflammatory effects through the Nrf2 pathway and the NLRP3 inflammasome, respectively [115]. Nevertheless, sulforaphane is not specific to the NLRP3 inflammasome since it has shown inhibitory activity for the AIM2 or NLRC4 inflammasome and NF-κB activation [137].

Cordycepin, a natural product derived from the medicinal fungus Cordyceps militaris, possesses various pharmacological properties, including tumor inhibition, suppression of inflammation and immunomodulation [138,139,140]. In a macrophage cell line, cordycepin notably inhibited LPS-induced activation of the NLRP3 inflammasome and the ERK1/2 signaling pathway [141]. In cerulein-induced AP in mice, cordycepin protected against pancreatic inflammatory processes by directly inhibiting NF-κB and NLRP3 inflammasome activation via AMPK [116].

#### 4.2.3. Non-Steroidal Anti-Inflammatory Drugs and Other Antioxidants 

The detrimental effects of oxidative stress and excessive inflammatory cascade reaction in the pathogenesis of AP have been extensively investigated [142]. Recent studies have suggested that cyclo-oxygenase-2 (COX-2) inhibitors are potent modulators of the inflammatory response and NLRP3 inflammasome activation [143]. Thus, the use of non-steroidal anti-inflammatory drugs (NSAIDs) appears a viable option for inflammasome inhibition.

Indomethacin, one of the most common NSAIDs and COX-2 inhibitors, has an evident anti-inflammatory effect and is widely used in rheumatoid arthritis and other diseases [144,145]. Clinical experience with NSAIDS for treating AP has centered mostly on the prevention of post-endoscopic retrograde cholangiopancreatography pancreatitis and has shown that patients who received indomethacin were less likely to develop this form of pancreatitis [146]. In an experimental AP model induced by cerulein combined with LPS in mice, indomethacin has recently been found to protect against pancreatic damage through inhibition of the NLRP3 inflammasome components [117]. 

Another selective COX-2 inhibitor, Iguratimod, has been reported to play an anti-inflammatory role against cerulein plus LPS-induced AP in mice through inhibition of the NF-κB signaling pathway and NLRP3 inflammasome activity [118].

The use of apocynin, an inhibitor of NADPH oxidase, mitigated AP-induced acute lung injury as well as pancreatic injury [119]. The mechanism underlying these protective effects includes the inhibition of the activation of NLRP3 inflammasome associated proteins NLRP3, pro-caspase-1 and IL1β.

#### 4.2.4. Bile Acids

In addition to their role as metabolic regulators, bile acids are involved in the modulation of inflammatory responses and the maintenance of immune homeostasis [147]. INT-777, a bile acid receptor agonist, has a protective effect against many inflammatory diseases [148]. Recent studies have found that INT-777 plays a regulatory role in the ROS/NLRP3 signaling. In the context of AP, INT-777 effectively alleviated the inflammation and pancreatic acinar cell injury by blockading the ROS/NLRP3 pathway [120].

#### 4.2.5. Antibiotics

Dysfunctional intestinal homeostasis in the early stage of AP has been shown to enhance intestinal bacterial translocation, subsequently exacerbating AP. A very recent publication by Jia et al. revealed the beneficial effect of prophylactic antibiotic treatment on AP development [149]. In a mouse model of cerulein-induced AP, combinatorial antibiotic treatment reduced pancreatic inflammation and decreased gut bacteria translocation to the pancreas through colonic TLR4/NLRP3 inflammasome inhibition. This procedure improved pancreatic inflammation and delayed the progression of AP into a systemic inflammatory response. 

## 5. Concluding Remarks and Perspectives

In 1925, Lord Moynihan’s description of AP reflected its dramatic nature: “the most terrible of all calamities that occurs in connection with the abdominal viscera. The suddenness of its onset, the illimitable agony which accompanies it, and the mortality attendant upon it, all renders it the most formidable of catastrophes”. Almost a century later, AP continues to be a devastating gastrointestinal disease with high morbidity and mortality, relatively little scope for action, and an often unpredictable outcome. Fundamental insights into the pathophysiology of AP have notably increased our knowledge of this disease in recent years, but there are still no effective treatment options available. The definition of early disease management strategies would help to mitigate the associated systemic complications, which are the main cause of death in these patients.

No studies have explored the clinical benefits of NLRP3 inhibition in patients with AP. We do know, however, that the levels of NLRP3 and AIM2 inflammasomes are increased in the early course of AP [41]; therefore, clinical studies that investigate the ability of inflammasome activation to influence AP development and progression are now urgently needed.

A role for IL1β in the inflammasome pathway has been clearly identified in a range of sterile inflammatory human diseases. IL1β is regarded as the major driver of inflammasome-mediated inflammation and so the currently available clinical treatment for NLRP3-related diseases use agents that target this cytokine. The effectiveness of IL1R blockade firmly establishes the value of using additional therapeutics that target the IL1 family of cytokines. Thus, it would be interesting to determine whether addition of IL1 antagonists other than IL1β might prove beneficial in the treatment of AP. In addition, as some cytokines have the ability to compensate the absence of others, combining IL1β and IL18 blockades, for example, may be of interest in order to enhance efficiency.

Inflammasome signaling can also affect biological processes other than inflammatory cytokine production. Among the upstream mechanisms that trigger NLRP3 activation, HMGB1 is of particular interest. HMGB1-targeted therapy has proved highly successful in numerous preclinical experimental models of inflammation but no clinical trials with HMGB1-specific agents have yet been conducted. In severe AP, circulating HMGB1 levels broadly reflect the severity of the disease. In addition, extracellular HMGB1 induces further local pancreatic and systemic injury; therefore, targeting HMGB1 could be an interesting therapeutic approach for AP and other sterile inflammatory conditions.

Moreover, the post-translational modifications of NLRP3 have emerged as important mechanisms in the control of its assembly. Research on the identification of the unknown factors controlling these modifications may help to identify novel pharmacological strategies for inhibiting inflammasome activation.

Collectively, given the vital importance of the inflammatory response in AP, further in-depth studies of the inflammasome are now needed with the aim of developing treatments for this disease. The fact that the NLRP3 inflammasome relies on numerous regulatory mechanisms may open up a whole range of therapeutic possibilities.

## Figures and Tables

**Table 1:** Inhibitory compounds of NLRP3 inflammasome activation in acute pancreatitis.

Compound	Type	Target	References
MCC950	Diarylsulphonylurea	NLRP3 (ASC oligomerization)	[42]
Glyburide	Sulphonylurea	NLRP3 (ATP-sensitive K^+^ channels)	[108]
Emodin	Anthraquinone	Nrf2/ NF-κB/ NLRP3/ P2X7	[109,110]
Danshensu	Phenolic acid	NF-κB/ STAT3/ NLRP3	[111]
Fraxinellone	Limonoid	NLRP3 (CARD, caspase-1, IL1β, IL18)	[112]
Withaferin A	Alkaloid	NF-κB/ NLRP3	[113]
Rutin	Flavonoid	NLRP3 (ASC, caspase-1)	[114]
Sulforaphane	Isothiocyanate	Nrf2/ NLRP3	[115]
Cordycepin	Adenosine analogue	NF-κB/NLRP3	[116]
Indomethacin	COX-2 inhibitor	NLRP3 (ASC, IL1β)	[117]
Iguratimod	COX-2 inhibitor	NF-κB/ NLRP3	[118]
Apocynin	NOX inhibitor	NF-κB/ NLRP3	[119]
INT-777	Bile acid receptor agonist	ROS/ NLRP3	[120]

NLRP3, NLR pyrin domain-containing protein 3; ASC, caspase recruitment domain; CARD, caspase activation and recruitment domain; NF-κB, nuclear factor kappa B; Nrf2, nuclear factor erythrocyte-2 associated factor-2; COX2, cyclo-oxygenase-2; NOX, NADPH oxidase; ROS, reactive oxygen species.

## Abbreviations

AIM	Absence in melanoma 2
ALRs	Absence in melanoma 2-like receptors
AP	Acute pancreatitis
ASC	Caspase recruitment domain
CAPS	Cryopyrin-associated periodic syndrome
CARD	Caspase activation and recruitment domain
CARS	Compensatory anti-inflammatory response syndrome
CLRs	C-type lectin receptors
COX-2	Cyclo-oxygenase-2
DAMPs	Damage-Associated Molecular Patterns
GSDM-D	Gasdermin-D
HMGB1	High mobility group box 1
IL	Interleukin
IL1R	Interleukin 1 receptor
IRAK	IL1R-associated kinase
IRF	Interferon regulatory factor
LPS	Lipopolysaccharide
MAPK	Mitogen-activated protein kinase
MyD88	Myeloid differentiation primary response 88
NATCH	Nucleotide-binding and oligomerization domain
NF-κB	Nuclear factor-kappa B
NLRP3	NLR pyrin domain containing protein 3
NOD1	NLR nucleotide-binding oligomerization domain-containing protein 1
Nrf2	Nuclear factor erythrocyte-2 associated factor 2
NSAIDS	Non-steroidal anti-inflammatory drugs
PAMPs	Pathogen-Associated Molecular Patterns
PRR	Pattern recognition receptors
PYD	Pyrin domain
RAGE	Receptor for advanced glycation end-products
RLRs	Retinoic acid-inducible gene (RIG)-I-like receptors
ROS	Reactive oxygen species
SIRS	Systemic inflammatory response syndrome
TLR	Toll-like receptor
TNFα	Tumor necrosis factor alpha
TRAF	Tumor necrosis factor receptor-activated factor
TRIF	TIR-domain-containing adaptor molecule inducing interferon-beta
